# Pathological narcissism and loneliness among men: Implications for men's satisfaction with life

**DOI:** 10.1111/papt.70056

**Published:** 2026-03-12

**Authors:** Georgia de Rappard‐Yuswack, David Kealy, John L. Oliffe, John S. Ogrodniczuk

**Affiliations:** ^1^ Department of Psychiatry University of British Columbia Vancouver British Columbia Canada; ^2^ School of Nursing University of British Columbia Vancouver British Columbia Canada

**Keywords:** loneliness, masculinity, men, narcissism, satisfaction with life

## Abstract

**Objectives:**

Loneliness has been established as a threat to health and well‐being, and may be particularly problematic for men due to masculinity norms that emphasize self‐reliance. Given the importance of understanding loneliness and its threat to well‐being among men, the present study was designed to examine a dispositional risk factor, pathological narcissism, on men's experience of loneliness and reduced satisfaction with life.

**Design:**

An online prospective cohort design was used to examine relations between pathological narcissism, including narcissistic vulnerability and grandiosity, loneliness and life satisfaction.

**Methods:**

A sample of men seeking online mental health help was recruited to complete self‐report measures regarding pathological narcissism, loneliness and life satisfaction across a six‐month period. Linear regression analyses were used to examine men's (*N* = 319, *M*age = 38.85 + 14.39) narcissistic vulnerability and grandiosity in relation to loneliness and to test mediation models whereby loneliness (at 3 months) would mediate the relationship between narcissistic vulnerability (baseline) and life satisfaction (at six months), including variability in the latter by controlling for baseline life satisfaction.

**Results:**

When examined together, only narcissistic vulnerability and not grandiosity was significant in predicting loneliness. Mediation models indicated significant indirect effects of baseline narcissistic vulnerability on life satisfaction at six months—and change in life satisfaction over six months—through the mediating effect of interim loneliness.

**Conclusions:**

Findings indicate narcissistic vulnerability as a significant dispositional factor in men's loneliness, and that such loneliness in turn diminishes men's general well‐being.

**Practitioner Points**

- Men with higher levels of narcissistic vulnerability are more likely to experience problems with loneliness, and such loneliness accounts for men's later sense of reduced satisfaction with life.
- For men seeking psychological help for reduced life satisfaction, it may be important for clinicians to evaluate narcissistic vulnerability and consider interventions that address this kind of dysregulated self‐image as well as associated loneliness.

## INTRODUCTION

Loneliness, the pain arising from inadequate social connection, is a major risk factor for poor health (Cacioppo et al., 2015). Meta‐analyses link loneliness to mood, substance use, cognitive, cardiovascular and immune disorders as well as increased all‐cause mortality (Cacioppo et al., 2015; Park et al., 2020; Rico‐Uribe et al., 2018; Valtorta et al., 2016). Men who report a greater degree of loneliness also tend to feel less pleasure and meaning in life, viewing life overall as less satisfying (Borawski, 2021; Mellor et al., 2008). Thus, loneliness may be an important contributor to overall reduced well‐being and discontentment with life.

There is growing evidence to suggest that the impact of loneliness on men's mental health is particularly salient (Ernst et al., 2021; Kealy et al., 2021; Keum et al., 2023; Zebhauser et al., 2014). Masculinity norms, including emphasis on self‐reliance, have been identified as potentially contributory to men's loneliness (Nordin et al., 2024). However, loneliness is a complex social phenomenon and there is a need for further empirical understanding of the factors that influence loneliness in men. Indeed, while reduced social engagement may be implicated, loneliness is more than aloneness; it can be experienced within social relationships when men perceive the quality of their relationships as inadequate (Qualter et al., 2015). Given the complex and subjective nature of loneliness, it is important to examine how dispositional factors, including personality, may interact with men's loneliness.

One potential contributor to men's loneliness and well‐being may be an aspect of personality known as pathological narcissism. Pathological narcissism is associated with significant challenges in mood, self‐esteem and interpersonal functioning (Brailovskaia et al., 2021; Finch & Kealy, 2024; Kealy et al., 2022; Pincus & Lukowitsky, 2010). This dimensional personality constellation is organized around impairment in and maladaptive regulation of self‐image, often involving a pattern of grandiosity, entitlement, blunted empathy and a deep need for admiration (Pincus & Lukowitsky, 2010). Pathological narcissism can present with diverse maladaptive coping mechanisms that are often categorized into narcissistic grandiosity or narcissistic vulnerability (Stinson et al., 2008). Narcissistic grandiosity describes features of arrogance, dominance, self‐enhancing behaviour, with strongly adverse responses to rejection, and the potential to be exploitative of others or even violent (Wright & Edershile, 2018). Narcissistic vulnerability describes an alternative way of coping with a fragile self‐image, including avoidance, shyness, hypervigilance to potential rejection and intense shame, alongside a deep sense of entitlement that may evoke envy (Wright & Edershile, 2018).

High levels of pathological narcissism are associated with significant psychosocial dysfunction (Dashineau et al., 2019). The most severe form of narcissism, narcissistic personality disorder (NPD), has a high prevalence of psychiatric comorbidities and an increased risk of suicidality (Ansell et al., 2015; Giner et al., 2013; Stinson et al., 2008). Although NPD is more commonly diagnosed in men, a meta‐analysis examining clinical and subclinical measures of narcissism indicated that men tend to score slightly higher in narcissistic grandiosity, but that there are no sex differences in narcissistic vulnerability (Grijalva et al., 2015).

Recent research on men's loneliness identifies trends that have theoretical implications for the role of pathological narcissism (Kealy et al., 2021; Keum et al., 2023). One proposed precipitant of loneliness is an unwillingness to voice negative emotions or reveal personal vulnerabilities, often referred to as distress non‐disclosure or self‐concealment. Low levels of distress disclosure are associated with loneliness in men, particularly young men (Kealy et al., 2021; Keum et al., 2023). Reluctance to express vulnerabilities is often hypothesized to relate to traditional masculine norms of unwavering stoicism and self‐reliance that are impressed upon many men through various social mechanisms (Berke et al., 2018). Yet, concealment of perceived flaws and vulnerabilities is also characteristic of pathological narcissism. Moreover, men with narcissistic vulnerability can have a high sensitivity to criticism and thus may attribute greater importance to living up to masculine norms, preventing them from voicing their needs and emotions. Indeed, narcissistic vulnerability is characterized by contingent self‐esteem—a reliance on external feedback to preserve a relatively positive self‐image—potentially heightening men's vigilance to others' impressions regarding their masculine presentation and behaviours. Concurrently, an enhanced self‐image may be upheld by tightly adhering to the traditional masculine mould. Dominant and antagonistic behaviours—aspects of narcissistic grandiosity that for some men may reflect a distorted form and defence of their masculinity—may repel lasting social connections. Thus, many narcissism‐related dynamics could interfere with men's social connectedness and positive interpersonal relations. As a result, there may be greater levels of loneliness and, in turn, reduced well‐being, among men with higher levels of pathological narcissism.

According to recent research utilizing measures of pathological narcissism (e.g., Pathological Narcissism Inventory; Pincus et al., 2009), loneliness appears to be significantly associated with narcissistic dysfunction. Kealy et al. (2022) reported a positive association between pathological narcissism and loneliness in a community sample of young adults. Even when accounting for the effect of the Big Five personality traits (Openness, Conscientiousness, Extraversion, Agreeableness and Neuroticism), the association remained significant between loneliness and narcissistic vulnerability, but not grandiosity. Subsequent work, in a community sample, found that as participants high in pathological narcissism reported greater levels of loneliness, they were more likely to endorse interpersonal challenges, such as rejection sensitivity and aggression, and higher levels of psychological distress (Kealy et al., 2023). Finch and Kealy (2024) went on to highlight impairments in intimacy, defined as patterns of poor relationship depth and durability, as the main element of personality dysfunction that links loneliness and pathological narcissism. Again, there was a stronger association with loneliness and narcissistic vulnerability than grandiosity.

Considering the theoretical and empirical connections among loneliness, pathological narcissism and life satisfaction, it seems reasonable to hypothesize that pathological narcissism may lead to lower life satisfaction through experiences of loneliness. Only one previous mixed‐sex study directly examined loneliness as a mediator of the effect of pathological narcissism on life satisfaction. Kealy et al. (2022) found narcissistic vulnerability to be positively associated with loneliness and that loneliness mediated the relation between narcissistic vulnerability and low life satisfaction. It should be noted that their cross‐sectional sample (*N* = 120) was primarily female (74%) and composed entirely of university‐aged participants. Thus, further investigation, particularly across time and among a larger sample of men, could strengthen confidence in inferences regarding the narcissism–loneliness–life satisfaction pathway. If research can more clearly outline the indirect effects of narcissism—and more specifically the roles of vulnerability and grandiosity—on men's well‐being, along with the mediating effects of loneliness, then clinicians might more confidently target these issues in psychotherapy with men.

The purpose of the present study was to investigate associations between dimensions of pathological narcissism, loneliness and subsequent satisfaction in life among men. Specifically, and on the basis of previous research, narcissistic vulnerability—rather than or more so than grandiosity—was hypothesized to be positively associated with loneliness at three months and negatively associated with men's life satisfaction at six months. Moreover, loneliness was hypothesized to mediate the relationship between narcissistic vulnerability and satisfaction with life across a six‐month period. We also explored this mediated pathway with regard to variability in life satisfaction over time—change in life satisfaction over the intervening six months—by controlling for men's baseline ratings of life satisfaction.

## METHODS

### Participants and procedures

Participants were help‐seeking men visiting the HeadsUpGuys website (https://headsupguys.org) for information about mental health issues, who consented to participate in a survey study linked to the site. Based in Canada, HeadsUpGuys is a world‐leading global online resource for men's mental health, offering information about mental health challenges and suicidality alongside suggestions for self‐initiated lifestyle interventions and professional services (Ogrodniczuk et al., 2018, 2021). Men using this resource often do so to obtain help in the context of emotional or social obstructions to their satisfaction with life. Ethics approval for the study was granted by the Behavioural Research Ethics Board at the University of British Columbia (H17‐01334). Men visiting HeadsUpGuys who were interested in participating were linked to an independent survey site, hosted by Qualtrics, where they were provided with the informed consent page. Eligible participants were adults (≥18 years old) who self‐identified as men and were able to read and understand English. No exclusion criteria were specified. Those providing informed consent to participate then completed the survey online. Following the initial survey, participants were invited to consider completing further surveys at subsequent 3‐ and 6‐month time points.

The baseline survey was completed by a larger initial sample of 3769 men. Since a longitudinal design was employed for the present study, data were selected regarding men who completed surveys at all three time points, yielding a sample of 319 men. These men had an average age of 38.85 (*SD* = 14.39; range = 18–77). The majority of the sample, 75.5% (*n* = 241), identified as heterosexual; 13.2% (*n* = 42) identified as gay and 9.4% (*n* = 30) as bisexual. Regarding ethnicity, the majority, 83.4% (*n* = 266), reported being Caucasian. Most participants resided in Canada, 60.2% (*n* = 192), or the United States, 15.7% (*n* = 50). Respondents were married or in committed relationships (48.3%; *n* = 154); single (43.5%; *n* = 139); or separated/divorced (8.1%; *n* = 26).

### Measures

#### Pathological narcissism

Dimensions of pathological narcissism were assessed at baseline using the Super Brief Pathological Narcissism Inventory (SB‐PNI; Schoenleber et al., 2015). This 12‐item self‐report instrument measures narcissistic grandiosity and vulnerability (Pincus et al., 2009; Wright et al., 2010). Six items refer to grandiosity (e.g., ‘I often fantasize about performing heroic deeds’) and six items refer to vulnerability (e.g., ‘When people don't notice me, I start to feel bad about myself’). Items are scored using a 6‐point scale from 0 (*not at all like me*) to 5 (*very much like me*). Higher scores—the mean of items—indicate a greater degree of pathological narcissism. The SB‐PNI demonstrated good internal consistency in the present sample, with alpha coefficients of .79 for grandiosity and .80 for vulnerability.

#### Loneliness

The three‐item version of the UCLA Loneliness Scale (Hughes et al., 2004) was employed to assess loneliness at the three‐month time point. The scale comprises the following questions: ‘How often do you feel that you lack companionship?’ ‘How often do you feel left out?’ ‘How often do you feel isolated from others?’ Responses consist of 1 (*hardly ever*), 2 (*sometimes*) and 3 (*often*), with higher total scores indicating a greater degree of loneliness. Good internal consistency was observed in the present sample, with an alpha coefficient of .86.

#### Satisfaction with life

A single‐item rating of satisfaction with life was used at baseline and at the six‐month time point. This item was worded as a question, ‘In general, how satisfied are you with your life?’ and rated with a four‐point scale anchored by 1 (*very dissatisfied*) and 4 (*very satisfied*). Similar single‐item ratings of life satisfaction have been widely used in well‐being surveys (VanderWeele et al., 2020) and treatment studies (Kealy et al., 2020), and research has demonstrated the performance of similarly‐worded items to be on par with multi‐item measures of satisfaction with life (Cheung & Lucas, 2014).

### Analytic approach

Statistical analyses were conducted with SPSS version 29. Analyses included descriptive statistics and computation of zero‐order correlations among main study variables, followed by linear regression used to examine narcissistic grandiosity and vulnerability as predictors of loneliness and life satisfaction. Linear regression mediation models were also computed using the PROCESS macro version 5.0 (Hayes, 2022), examining the indirect effect of pathological narcissism (baseline) on life satisfaction (at six months) with loneliness (at three months) as a mediator, and with baseline life satisfaction and loneliness as covariates in the second model. These models employed bootstrapped 95% confidence intervals (CIs), at 10,000 re‐samples, to test the significance of the estimated indirect effect (i.e., mediation); an absence of zero within the CI indicates statistical significance at *p* < .05.

## RESULTS

To evaluate potential differences between men who completed the study and those who did not (i.e., the original sample of 3769 men), non‐parametric tests (Mann–Whitney U‐tests and chi‐square tests) were computed for several demographic characteristics and baseline pathological narcissism. These analyses found no significant differences regarding age (*p* = .92), pathological narcissism (*p* = .16), whether employed or not employed (*p* = .49) and whether in a committed relationship or not (*p* = .18). Descriptive statistics and zero‐order correlations are shown in Table 1. Significant correlations were observed among all study variables in expected directions, with both narcissism dimensions positively associated with loneliness and negatively associated with life satisfaction. Thus, linear regression analyses included both dimensions as predictors in order to examine their relative effects when holding each constant. The regression model predicting loneliness was significant, *F*(2, 316) = 41.17, *p* < .001, with narcissism accounting for 21% of the variance in loneliness, yet with only narcissistic vulnerability remaining a significant predictor (Table 2). The model predicting life satisfaction was also significant, *F*(2, 316) = 18.87, *p* < .001, with narcissism accounting for 11% of the variance. Similarly, only narcissistic vulnerability was a significant predictor of life satisfaction when both dimensions were included (Table 2).

**TABLE 1:** Descriptive statistics and zero‐order correlations regarding pathological narcissism, loneliness and satisfaction with life among men (*N* = 319).

	*M* (*SD*)	1	2	3
1. Narcissistic grandiosity (baseline)	2.89 (1.10)	—		
2. Narcissistic vulnerability (baseline)	2.56 (1.20)	.57**	—	
3. Loneliness (3 months)	6.71 (2.01)	.30**	.45**	—
4. Satisfaction with life (6 months)	2.39 (.91)	−.19**	−.33**	−.53**

** *p* < .001.

**TABLE 2:** Linear regression models examining narcissistic grandiosity and vulnerability as predictors of loneliness and satisfaction with life among men.

	*R* ^2^	*B*	*SE*	LLCI	ULCI	*β*	*t*	*p*
Predicting loneliness (3 months)	.207							
Narcissistic grandiosity		.111	.112	−.110	.331	.060	.987	.324
Narcissistic vulnerability		.700	.102	.498	.899	.418	6.847	<.001
Predicting satisfaction with life (6 months)	.107							
Narcissistic grandiosity		−.004	.054	−.110	.101	−.005	−.081	.936
Narcissistic vulnerability		−.244	.049	−.340	−.148	−.324	−5.000	<.001

Abbreviations: LLCI, lower limit, 95% confidence interval; ULCI, upper limit, 95% confidence interval.

**FIGURE 1:**
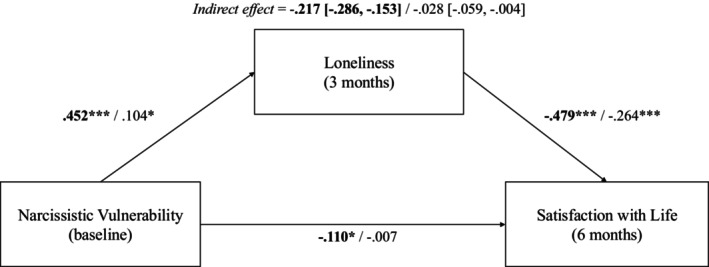
Standardized coefficients for regression models testing loneliness as a mediator of the relationship between narcissistic vulnerability and satisfaction with life. Unadjusted coefficients are in bold text; adjusted coefficients refer to controlling for baseline loneliness and satisfaction with life. *p < .05; **p < .001.

Given these findings, narcissistic vulnerability was subsequently examined as the independent variable in our hypothesized mediation models. The first model, with loneliness as mediator and life satisfaction as the dependent variable, was significant, *F*(2, 316) = 64.32, *p* < .001, with significant paths to loneliness at three months (*β* = −.46, *p* < .001) and life satisfaction at six months (*β* = .55, *p* < .001). Narcissistic vulnerability and loneliness accounted for 29% of the variance in life satisfaction at the 6‐month time point. As shown in Figure 1, a significant indirect effect was observed for narcissistic vulnerability on life satisfaction through the mediating effect of loneliness, indicated by a standardized point estimate of −.22, with a 95% CI [−.29, −.15] not containing zero. The size of this indirect effect suggests that a 1*SD* increase in narcissistic vulnerability would correspond to loneliness accounting for a .22*SD* reduction in life satisfaction.

The second model, examining variability in life satisfaction over six months, included baseline life satisfaction, *M* = 2.18, *SD* = .90 and baseline loneliness, *M* = 7.04, *SD* = 1.92, as covariates. This model was also significant, *F*(4, 314) = 73.71, *p* < .001, with predictors, mediators and covariates together accounting for 48% of the variance. Controlling for baseline loneliness and life satisfaction—thereby modelling variability across three and six months, respectively—resulted in reduced effect sizes across the model (Figure 1). However, the indirect effect of narcissistic vulnerability remained significant, with a standardized point estimate of −.028, 95% CI [−.059, −.005]. Thus, narcissistic vulnerability—through the mediating effect of variability in loneliness—was an indirect predictor of men's variability in life satisfaction over a six‐month period.

## DISCUSSION

This study examined dimensions of pathological narcissism among men in relation to their experience of loneliness and subsequent level of life satisfaction. At the level of zero‐order correlations, significant associations were found between both narcissistic grandiosity and narcissistic vulnerability and loneliness and life satisfaction. However, when examined simultaneously in multivariate regression, narcissistic grandiosity no longer exerted a significant effect. Thus, between these two domains, narcissistic vulnerability—including self‐concealment and contingent self‐esteem—was the main driver of men's loneliness and life satisfaction. Narcissistic vulnerability was also found to exert an indirect effect on subsequent life satisfaction (at six months) through the mediating effect of loneliness (at three months). Men with higher levels of narcissistic vulnerability were more likely to report a greater sense of loneliness, which in turn was associated with lower levels of subsequent satisfaction with life. Moreover, this indirect effect of narcissistic vulnerability—and the mediating effect of loneliness—was also significant when controlling for baseline loneliness and life satisfaction, indicating the salience of this pathway for changes in men's satisfaction with life over a six‐month period.

The mediation model tested in the present study is congruent with previous findings, in a community sample of young adults, regarding loneliness as a mediator of the relationship between narcissistic vulnerability and life satisfaction (Kealy et al., 2022). Our results not only bolster confidence in this pathway but also newly support a temporal aspect, wherein narcissistic vulnerability predicts changes in life satisfaction over time through loneliness as a mediator. This suggests that life satisfaction can be negatively influenced by higher levels of narcissistic vulnerability through increased loneliness. Validating this model in a large sample of men is noteworthy because of the detrimental effect of loneliness on men's mental health (Ernst et al., 2021; Zebhauser et al., 2014). This warrants consideration of whether aspects of masculine socialization impact men's loneliness experiences. Below, we consider why narcissistic vulnerability appears to promote loneliness and low life satisfaction in men, while speculating on the potential contribution of restrictive masculine norms.

In narcissistic vulnerability, unmet needs or wishes for validation are concealed by avoidant behaviour and potentially even confessions of low self‐esteem, suffering or helplessness (Pincus et al., 2009, 2014). In contrast to narcissistic grandiosity, which can present in ways that demand a supply of social attention like arrogance and declarations of unfounded achievement, narcissistic vulnerability captures qualities that might render social attention intolerable, invoking shame and hypersensitivity (Pincus et al., 2009, 2014). We found narcissistic vulnerability to have a stronger association with loneliness than grandiosity, with the effect of the latter being non‐significant when both dimensions were tested simultaneously in relation to loneliness. Thus, our findings suggest that men's self‐concealment and self‐worth being contingent on others' feedback is more predictive of loneliness than their reliance on grandiose fantasizing to support a deficient sense of self. Although the potential protective quality of grandiosity has been noted in other works (Carone et al., 2023; Dashineau et al., 2019; Pincus & Lukowitsky, 2010), fantasies of extraordinary achievement are likely an inadequate salve for negative beliefs underlying contingent self‐esteem, such as a sense of being unworthy or unlovable (Huprich & Malone, 2022; Kealy & Ogrodniczuk, 2014).

Theory connecting narcissistic vulnerability and loneliness has focused on distorted perceptions in social interactions and negative internal representations of self in relation to others (Kealy & Ogrodniczuk, 2014). It may be that avoidant patterns of interaction—a restriction in the capacity for close connectedness—as well as being perceived by others as cold or uninterested, could contribute to loneliness in the context of narcissistic vulnerability (Finch & Kealy, 2024). Additionally, narcissistic vulnerability is linked to a tendency to perceive others as harsher (Edershile & Wright, 2021). Impeded socializing may be exacerbated by hypervigilance to rejection, a feature of narcissistic vulnerability, but also a cognitive consequence of loneliness (Cacioppo et al., 2015). A negative feedback loop may arise, starting with vulnerability‐related hypervigilance to rejection that disincentivizes socialization, in turn leading to loneliness that further enhances the hypervigilance. These compounding behaviours and interpretations may culminate in men with higher levels of narcissistic vulnerability feeling isolated, stuck and dejected about their lives. There may also be an unwillingness to seek social connection to soothe this distress when one is fixated on the hostility of others.

Beyond such interpersonal barriers to socializing, other features of narcissistic vulnerability may further interfere with the degree of connectedness in close relationships. In narcissistic vulnerability, there is reluctance to express needs due to feelings of shame (Pincus & Lukowitsky, 2010). Similarly, men who strongly endorse traditional masculine ideologies (TMI) (e.g., self‐reliance, dominance, stoicism) have a lower tendency to express negative emotions (Jakupcak et al., 2003). Young men may be exposed to TMI through modelling and reinforcement (Addis et al., 2010; Berke et al., 2018), and the contingent self‐esteem in men high in narcissistic vulnerability may make them particularly wary of violating masculine norms, such as the proscription of emotional disclosure and dependency. Emotional concealment can be beneficial at times; however, it may contribute to loneliness and reduced life satisfaction if it persists across contexts where emotional expression is advantageous, such as in relationship building and therapy. Notably, reluctance to express negative emotions is associated with loneliness in young men in particular (Kealy et al., 2021; Keum et al., 2023) and strong adherence to TMI is associated with more anxiety and depressive symptoms (Genuchi et al., 2025; Montiel et al., 2022). A preoccupation with concern that others dislike them—an aspect of contingent self‐esteem (Pincus et al., 2009)—may intensify and complicate concerns over masculinity adherence. For example, a man may have a strong desire to prove his masculinity, seeking external validation to bolster his self‐image, yet he may simultaneously experience shame for being deficient and dependent on others. A man with higher levels of narcissistic vulnerability may feel paralysed by these discordant dynamics, leading to diminished social engagement and a sense of frustration and hopelessness about genuine connectedness, belonging and overall well‐being.

In addition to the aforementioned internal psychological processes—hypervigilance, shame and contingent self‐esteem—maladaptive behaviours that arise in response to perceived interpersonal threats, such as devaluation and aggression, may increase loneliness in narcissistic vulnerability (Besser & Priel, 2010; Day et al., 2022; Wright et al., 2017). For example, individuals with narcissistic vulnerability may have unusually high expectations for validation in relationships and may blame or criticize their partner when these perceived needs are not met (Szücs et al., 2020; Wright et al., 2017). Notably, narcissistic vulnerability, more so than grandiosity, is associated with dissatisfaction in current romantic relationships (Balzen et al., 2022). Seemingly, intimate partner relationships may not be sufficient to prevent loneliness in someone with high levels of narcissistic vulnerability, as they may always find fault in a partner and thus struggle to feel connected. Pathological narcissism is also associated with aggression that could promote loneliness and low life satisfaction through pushing others away (Kealy, Ogrodniczuk, et al., 2017). Specifically, in men with a fragile self‐image, perceived failures to live up to masculine ideals could trigger aggressive behaviour (Berke et al., 2016). In terms of cascading effects, it is likely that these relationship dynamics and stressors will end many men's partnerships, adding another significant suicidality risk factor (Wilson et al., 2025). The negative effects of such distressed relationships on men's partners are also a key safety and concurrent treatment consideration.

Providing effective care for pathological narcissism in men is challenging due to tendencies towards poor retention in therapy and a paucity of empirical data to guide gender‐responsive treatments (Ellison et al., 2013; Kealy, Goodman, et al., 2017). Moreover, some therapists report feeling frustrated and disengaged with male patients high in narcissism, which can erode the therapeutic alliance (Tanzilli et al., 2017). Our findings contribute to the ongoing dialogue about how to address these challenges. We highlight loneliness as a risk factor for distress in men presenting with features of pathological narcissism. Identifying loneliness as a mediator between narcissistic vulnerability and life satisfaction suggests that focusing on loneliness may be impactful when men present to therapy with the self‐image dysregulation characteristic of narcissistic difficulties, and with feelings of being stuck and dissatisfied with life. Paying particular attention to how men experience and narrate themselves in relationships with others, including the therapist, may help clarify the sources of dysfunction both in session and in the patient's life.

Addressing pathological narcissism and loneliness among men is important not only for the individual but potentially also for those around them in the community. Men with higher levels of pathological narcissism are more likely to exhibit externalizing behaviours (i.e., substance use, violence, antisociality) (Berke et al., 2018; Kealy, Ogrodniczuk, et al., 2017), and loneliness appears to strengthen the link between narcissism and interpersonal aggression (Kealy et al., 2023); thus, loneliness may trigger domineering or vindictive behaviour (Cheek et al., 2018). For some men, this may manifest in the form of threatening child custody battles or physical violence in response to partners who request separation (Day et al., 2022). Thus, clinical attention to men's contingent self‐esteem, distorted social perceptions and diminished connectedness may have preventative value and impact for those around them.

The limitations of our study should be noted. Our results rely upon self‐report measures, which may be influenced by social desirability and self‐enhancement bias. General personality traits were not examined, so it is unclear whether other basic personality features might influence results, although when personality traits have been included in similar studies, the results align with our findings (Kealy et al., 2022). Nevertheless, assessment of pathological narcissism using self‐report methods may be particularly subject to bias because of the nature of narcissistic self‐image dysregulation. While some individuals with pathological narcissism may be reluctant to report experiences or personality features that challenge their own distorted self‐image, others may provide biased responses due to limited insight into their psychological functioning (Dashineau et al., 2019). Moreover, psychiatric diagnosis was not considered, nor the presence of co‐occurring psychological problems or disorders. These issues can also interact with narcissistic difficulties, adding to the challenges of research using self‐report assessment. The men in our sample were seeking information regarding mental health concerns, and may have commenced the study with lower levels of life satisfaction than non‐help‐seeking men, which may limit generalizability across broader populations of men. Another limitation involves our sample's relatively limited ethnic and cultural diversity; a large majority of the sample identified as Caucasian. Moreover, intervening social experiences—various types of which could influence loneliness and life satisfaction—were not accounted for in the present study. Finally, our study did not examine masculine norm adherence. Although the men in our sample likely experienced gender socialization to masculinity norms, our data do not allow for inferences regarding the nature of men's adherence to these norms as an interaction with pathological narcissism and loneliness. Future research could address these limitations by employing varied and more comprehensive assessment methods, diverse sampling, and inclusion of socialization variables, including adherence to traditional masculinity ideology. Methods such as ecological momentary assessment, aimed at capturing more fine‐grained fluctuation in loneliness and other social and emotional variables that were not considered in the present study, could also advance knowledge of men's loneliness and well‐being in relation to personality features.

In conclusion, the present study—strengthened by longitudinal data collection over three time points—indicated that men with higher levels of pathological narcissism experienced a greater degree of loneliness, an effect driven largely by narcissistic vulnerability, and subsequently lower life satisfaction. Moreover, the relationship between higher narcissistic vulnerability and decreased life satisfaction over a six‐month period was mediated by variability in loneliness. Thus, loneliness emerged as a key variable in understanding why men with unstable self‐image and self‐concealment tendencies characteristic of narcissistic vulnerability tend to have greater discontentment with life. These findings offer insight into salient negative internal psychological processes, such as contingent self‐esteem and shame, which are worth considering when men present to care with loneliness, reduced well‐being and/or features of personality dysfunction.

## AUTHOR CONTRIBUTIONS

**Georgia de Rappard‐Yuswack:** Writing – original draft; writing – review and editing. **David Kealy:** Conceptualization; formal analysis; writing – original draft; writing – review and editing; investigation; methodology. **John L. Oliffe:** Writing – review and editing. **John S. Ogrodniczuk:** Writing – review and editing; project administration; data curation.

## CONFLICT OF INTEREST STATEMENT

The authors declare no conflicts of interest.

## ETHICS STATEMENT

This study was approved by the Behavioural Research Ethics Board of the University of British Columbia, and the procedures followed were in accordance with the 2013 revised Helsinki Declaration. All participants provided informed consent to participate in this study.

## Data Availability

Data regarding this study may be made available to researchers upon reasonable request to the corresponding author.

## References

[papt70056-bib-0001] Addis, M. E. , Mansfield, A. K. , & Syzdek, M. R. (2010). Is “masculinity” a problem?: Framing the effects of gendered social learning in men. Psychology of Men & Masculinity, 11(2), 77–90. 10.1037/A0018602

[papt70056-bib-0002] Ansell, E. B. , Wright, A. G. C. , Markowitz, J. C. , Sanislow, C. A. , Hopwood, C. J. , Zanarini, M. C. , Yen, S. , Pinto, A. , McGlashan, T. H. , & Grilo, C. M. (2015). Personality disorder risk factors for suicide attempts over 10 years of follow‐up. Personality Disorders, 6(2), 161–167. 10.1037/per0000089 25705977 PMC4415153

[papt70056-bib-0003] Balzen, K. M. , Knoch, D. A. , Millward, K. A. , Corretti, C. A. , & Ackerman, R. A. (2022). Narcissistic traits and romantic relationship outcomes: A short daily diary investigation. Journal of Research in Personality, 96, 104179. 10.1016/j.jrp.2021.104179

[papt70056-bib-0004] Berke, D. S. , Reidy, D. , & Zeichner, A. (2018). Masculinity, emotion regulation, and psychopathology: A critical review and integrated model. Clinical Psychology Review, 66, 106–116. 10.1016/j.cpr.2018.01.004 29398184

[papt70056-bib-0005] Berke, D. S. , Reidy, D. E. , Gentile, B. , & Zeichner, A. (2016). Masculine discrepancy stress, emotion‐regulation difficulties, and intimate partner violence. Journal of Interpersonal Violence, 34(6), 1163–1182. 10.1177/0886260516650967 27226013 PMC5861012

[papt70056-bib-0006] Besser, A. , & Priel, B. (2010). Grandiose narcissism versus vulnerable narcissism in threatening situations: Emotional reactions to achievement failure and interpersonal rejection. Journal of Social & Clinical Psychology, 29(8), 874–902. 10.1521/JSCP.2010.29.8.874

[papt70056-bib-0007] Borawski, D. (2021). Authenticity and rumination mediate the relationship between loneliness and well‐being. Current Psychology, 40(9), 4663–4672. 10.1007/s12144-019-00412-9

[papt70056-bib-0008] Brailovskaia, J. , Bierhoff, H. W. , & Rohmann, E. (2021). Loneliness and depression symptoms: The moderating role of narcissism. Journal of Affective Disorders Reports, 6, 100264. 10.1016/j.jadr.2021.100264

[papt70056-bib-0009] Cacioppo, S. , Grippo, A. J. , London, S. , Goossens, L. , & Cacioppo, J. T. (2015). Loneliness: Clinical import and interventions. Perspectives on Psychological Science, 10(2), 238–249. 10.1177/1745691615570616 25866548 PMC4391342

[papt70056-bib-0010] Carone, N. , Benzi, I. M. A. , Parolin, L. A. L. , & Fontana, A. (2023). “I can't miss a thing” – The contribution of defense mechanisms, grandiose narcissism, and vulnerable narcissism to fear of missing out in emerging adulthood. Personality and Individual Differences, 214, 112333. 10.1016/j.paid.2023.112333

[papt70056-bib-0011] Cheek, J. , Kealy, D. , Joyce, A. S. , & Ogrodniczuk, J. S. (2018). Interpersonal problems associated with narcissism among psychiatric outpatients: A replication study. Archives of Psychiatry and Psychotherapy, 20(2), 26–33. 10.12740/app/90328

[papt70056-bib-0012] Cheung, F. , & Lucas, R. E. (2014). Assessing the validity of single‐item life satisfaction measures: Results from three large samples. Quality of Life Research, 23(10), 2809–2818. 10.1007/s11136-014-0726-4 24890827 PMC4221492

[papt70056-bib-0013] Dashineau, S. C. , Edershile, E. A. , Simms, L. J. , & Wright, A. G. C. (2019). Pathological narcissism and psychosocial functioning. Personality Disorders, 10(5), 473–478. 10.1037/per0000347 31259606 PMC6710132

[papt70056-bib-0014] Day, N. J. S. , Townsend, M. L. , & Grenyer, B. F. S. (2022). Pathological narcissism: An analysis of interpersonal dysfunction within intimate relationships. Personality and Mental Health, 16(3), 204–216. 10.1002/pmh.1532 34783453 PMC9541508

[papt70056-bib-0015] Edershile, E. A. , & Wright, A. G. C. (2021). Grandiose and vulnerable narcissistic states in interpersonal situations. Self and Identity, 20(2), 165–181. 10.1080/15298868.2019.1627241 33716581 PMC7953573

[papt70056-bib-0016] Ellison, W. D. , Levy, K. N. , Cain, N. M. , Ansell, E. B. , & Pincus, A. L. (2013). The impact of pathological narcissism on psychotherapy utilization, initial symptom severity, and early‐treatment symptom change: A naturalistic investigation. Journal of Personality Assessment, 95(3), 291–300. 10.1080/00223891.2012.742904 23186259

[papt70056-bib-0017] Ernst, M. , Klein, E. M. , Beutel, M. E. , & Brähler, E. (2021). Gender‐specific associations of loneliness and suicidal ideation in a representative population sample: Young, lonely men are particularly at risk. Journal of Affective Disorders, 294, 63–70. 10.1016/j.jad.2021.06.085 34274789

[papt70056-bib-0018] Finch, E. F. , & Kealy, D. (2024). Loneliness in narcissistic vulnerability: Examining domains of personality functioning. Personality and Mental Health, 18, 259–268. 10.1002/pmh.1615 38666522

[papt70056-bib-0019] Genuchi, M. C. , Oliffe, J. L. , Rice, S. M. , Kealy, D. , Walther, A. , Seidler, Z. E. , & Ogrodniczuk, J. S. (2025). Thought suppression strategies as mediators between traditional masculinity ideology and externalized depressive symptoms in men. Current Psychology, 44, 1–16. 10.1007/s12144-025-07542-3

[papt70056-bib-0020] Giner, L. , Blasco‐Fontecilla, H. , Mercedes Perez‐Rodriguez, M. , Garcia‐Nieto, R. , Giner, J. , Guija, J. A. , Rico, A. , Barrero, E. , Luna, M. A. , De Leon, J. , Oquendo, M. A. , & Baca‐Garcia, E. (2013). Personality disorders and health problems distinguish suicide attempters from completers in a direct comparison. Journal of Affective Disorders, 151(2), 474–483. 10.1016/j.jad.2013.06.029 23859005

[papt70056-bib-0021] Grijalva, E. , Newman, D. A. , Tay, L. , Brent Donnellan, M. , Harms, P. D. , Robins, R. W. , & Yan, T. (2015). Gender differences in narcissism: A meta‐analytic review. Psychological Bulletin, 141(2), 261–310. 10.1037/A0038231 25546498

[papt70056-bib-0022] Hayes, A. F. (2022). Introduction to mediation, moderation, and conditional process analysis: A regression‐based approach (3rd ed.). Guilford Press.

[papt70056-bib-0023] Hughes, M. E. , Waite, L. J. , Hawkley, L. C. , & Cacioppo, J. T. (2004). A short scale for measuring loneliness in large surveys: Results from two population‐based studies. Research on Aging, 26(6), 655–672. 10.1177/0164027504268574 18504506 PMC2394670

[papt70056-bib-0024] Huprich, S. K. , & Malone, B. C. (2022). Malignant self‐regard: Overview and future directions. Harvard Review of Psychiatry, 30(4), 226–237. 10.1097/hrp.0000000000000342 35849740

[papt70056-bib-0025] Jakupcak, M. , Salters, K. , Gratz, K. L. , & Roemer, L. (2003). Masculinity and emotionality: An investigation of men's primary and secondary emotional responding. Sex Roles, 49(3–4), 111–120. 10.1023/a:1024452728902

[papt70056-bib-0026] Kealy, D. , Aafjes‐van Doorn, K. , Ehrenthal, J. C. , Weber, R. , Ogrodniczuk, J. S. , & Joyce, A. S. (2020). Improving social functioning and life satisfaction among patients with personality dysfunction: Connectedness and engagement in integrative group treatment. Clinical Psychology & Psychotherapy, 27(3), 288–299. 10.1002/cpp.2427 31950590

[papt70056-bib-0027] Kealy, D. , Goodman, G. , Rasmussen, B. , Weideman, R. , & Ogrodniczuk, J. S. (2017). Therapists perspectives on optimal treatment for pathological narcissism. Personality Disorders, Theory, Research, and Treatment, 8(1), 35–45. 10.1037/per0000164 26595343

[papt70056-bib-0028] Kealy, D. , & Ogrodniczuk, J. S. (2014). Pathological narcissism and the obstruction of love. Psychodynamic Psychiatry, 42(1), 101–120. 10.1521/pdps.2014.42.1.101 24555464

[papt70056-bib-0029] Kealy, D. , Ogrodniczuk, J. S. , Rice, S. M. , & Oliffe, J. L. (2017). Pathological narcissism and maladaptive self‐regulatory behaviours in a nationally representative sample of Canadian men. Psychiatry Research, 256, 156–161. 10.1016/j.psychres.2017.06.009 28641202

[papt70056-bib-0030] Kealy, D. , Seidler, Z. E. , Rice, S. M. , Cox, D. W. , Oliffe, J. L. , Ogrodniczuk, J. S. , & Kim, D. (2021). Reduced emotional awareness and distress concealment: A pathway to loneliness for young men seeking mental health care. Frontiers in Psychology, 12, 679639. 10.3389/fpsyg.2021.679639 34234718 PMC8255362

[papt70056-bib-0031] Kealy, D. , Woolgar, S. , & Hewitt, J. M. A. (2022). Investigating pathological narcissism and loneliness, and the link with life satisfaction. Scandinavian Journal of Psychology, 63(1), 32–38. 10.1111/sjop.12773 34524693

[papt70056-bib-0032] Kealy, D. , Woolgar, S. , Hewitt, J. M. A. , & Cox, D. W. (2023). When narcissism gets lonely: Loneliness moderates the association between pathological narcissism and interpersonal problems, and the link to psychological distress. Current Psychology, 42(20), 17110–17119. 10.1007/s12144-022-02976-5

[papt70056-bib-0033] Keum, B. T. H. , Oliffe, J. L. , Rice, S. M. , Kealy, D. , Seidler, Z. E. , Cox, D. W. , Levant, R. F. , & Ogrodniczuk, J. S. (2023). Distress disclosure and psychological distress among men: The role of feeling understood and loneliness. Current Psychology, 42(13), 10533–10542. 10.1007/s12144-021-02163-y

[papt70056-bib-0034] Mellor, D. , Stokes, M. , Firth, L. , Hayashi, Y. , & Cummins, R. (2008). Need for belonging, relationship satisfaction, loneliness, and life satisfaction. Personality and Individual Differences, 45(3), 213–218. 10.1016/j.paid.2008.03.020

[papt70056-bib-0035] Montiel, A. , Quan, C. , & Costigan, C. L. (2022). No man is an Island: Associations between adherence to traditional masculine norms and young men's psychosocial adjustment. Canadian Journal of Behavioural Science, 55(1), 56–67. 10.1037/CBS0000345

[papt70056-bib-0036] Nordin, T. , Degerstedt, F. , & Granholm Valmari, E. (2024). A scoping review of masculinity norms and their interplay with loneliness and social connectedness among men in Western societies. American Journal of Men's Health, 18(6), 1–14. 10.1177/15579883241304585 PMC11626675 39651586

[papt70056-bib-0037] Ogrodniczuk, J. , Oliffe, J. , & Beharry, J. (2018). HeadsUpGuys: Canadian online resource for men with depression. Canadian Family Physician, 64(2), 93–94. https://pmc.ncbi.nlm.nih.gov/articles/pmc5964376/ 29449231 PMC5964376

[papt70056-bib-0038] Ogrodniczuk, J. S. , Beharry, J. , & Oliffe, J. L. (2021). An evaluation of 5‐year web analytics for HeadsUpGuys: A men's depression e‐mental health resource. American Journal of Men's Health, 15(6), 15579883211063322. 10.1177/15579883211063322 PMC8646842 34861812

[papt70056-bib-0039] Park, C. , Majeed, A. , Gill, H. , Tamura, J. , Ho, R. C. , Mansur, R. B. , Nasri, F. , Lee, Y. , Rosenblat, J. D. , Wong, E. , & McIntyre, R. S. (2020). The effect of loneliness on distinct health outcomes: A comprehensive review and meta‐analysis. Psychiatry Research, 294, 113514. 10.1016/j.psychres.2020.113514 33130511

[papt70056-bib-0040] Pincus, A. L. , Ansell, E. B. , Pimentel, C. A. , Cain, N. M. , Wright, A. G. C. , & Levy, K. N. (2009). Initial construction and validation of the pathological narcissism inventory. Psychological Assessment, 21(3), 365–379. 10.1037/a0016530 19719348

[papt70056-bib-0041] Pincus, A. L. , & Lukowitsky, M. R. (2010). Pathological narcissism and narcissistic personality disorder. Annual Review of Clinical Psychology, 6, 421–446. 10.1146/annurev.clinpsy.121208.131215 20001728

[papt70056-bib-0042] Pincus, A. L. , Wright, A. G. C. , & Cain, N. M. (2014). Narcissistic grandiosity and narcissistic vulnerability in psychotherapy. Personality Disorders, Theory, Research, and Treatment, 5(4), 439–443. 10.1037/per0000031 24446581

[papt70056-bib-0043] Qualter, P. , Vanhalst, J. , Harris, R. , Van Roekel, E. , Lodder, G. , Bangee, M. , Maes, M. , & Verhagen, M. (2015). Loneliness across the life span. Perspectives on Psychological Science, 10(2), 250–264. 10.1177/1745691615568999 25910393

[papt70056-bib-0044] Rico‐Uribe, L. A. , Caballero, F. F. , Martín‐María, N. , Cabello, M. , Ayuso‐Mateos, J. L. , & Miret, M. (2018). Association of loneliness with all‐cause mortality: A meta‐analysis. PLoS One, 13(1), e0190033. 10.1371/journal.pone.0190033 29300743 PMC5754055

[papt70056-bib-0045] Schoenleber, M. , Roche, M. J. , Wetzel, E. , Pincus, A. L. , & Roberts, B. W. (2015). Development of a brief version of the pathological narcissism inventory. Psychological Assessment, 27(4), 1520–1526. 10.1037/pas0000158 26011478 PMC4659764

[papt70056-bib-0046] Stinson, F. S. , Dawson, D. A. , Goldstein, R. B. , Chou, S. P. , Huang, B. , Smith, S. M. , Ruan, W. J. , Pulay, A. J. , Saha, T. D. , Pickering, R. P. , & Grant, B. F. (2008). Prevalence, correlates, disability, and comorbidity of DSM‐IV narcissistic personality disorder: Results from the wave 2 National Epidemiologic Survey on alcohol and related conditions. The Journal of Clinical Psychiatry, 69(7), 1033. 10.4088/jcp.v69n0701 18557663 PMC2669224

[papt70056-bib-0047] Szücs, A. , Szanto, K. , Adalbert, J. , Wright, A. G. C. , Clark, L. , & Dombrovski, A. Y. (2020). Status, rivalry and admiration‐seeking in narcissism and depression: A behavioral study. PLoS One, 15(12), e0243588. 10.1371/journal.pone.0243588 33270780 PMC7714187

[papt70056-bib-0048] Tanzilli, A. , Muzi, L. , Ronningstam, E. , & Lingiardi, V. (2017). Countertransference when working with narcissistic personality disorder: An empirical investigation. Psychotherapy, 54(2), 184–194. 10.1037/pst0000111 28581327

[papt70056-bib-0049] Valtorta, N. K. , Kanaan, M. , Gilbody, S. , Ronzi, S. , & Hanratty, B. (2016). Loneliness and social isolation as risk factors for coronary heart disease and stroke: Systematic review and meta‐analysis of longitudinal observational studies. Heart (British Cardiac Society), 102(13), 1009–1016. 10.1136/heartjnl-2015-308790 27091846 PMC4941172

[papt70056-bib-0050] VanderWeele, T. J. , Trudel‐Fitzgerald, C. , Allin, P. , Farrelly, C. , Fletcher, G. , Frederick, D. E. , Hall, J. , Helliwell, J. F. , Kim, E. S. , Lauinger, W. A. , Lee, M. T. , Lyubomirsky, S. , Margolis, S. , McNeely, E. , Messer, N. , Tay, L. , Viswanath, V. , Węziak‐Białowolska, D. , & Kubzansky, L. D. (2020). Current recommendations on the selection of measures for well‐being. Preventive Medicine, 133, 106004. 10.1016/j.ypmed.2020.106004 32006530

[papt70056-bib-0051] Wilson, M. J. , Scott, A. J. , Pilkington, V. , Macdonald, J. A. , Rice, S. M. , Oliffe, J. L. , & Seidler, Z. E. (2025). Suicidality in men following relationship breakdown: A systematic review and meta‐analysis of global data. Psychological Bulletin, 151, 819–860. 10.1037/bul0000482 40674014

[papt70056-bib-0052] Wright, A. G. , & Edershile, E. A. (2018). Issues resolved and unresolved in pathological narcissism. Current Opinion in Psychology, 21, 74–79. 10.1016/j.copsyc.2017.10.001 29059578

[papt70056-bib-0053] Wright, A. G. C. , Lukowitsky, M. R. , Pincus, A. L. , & Conroy, D. E. (2010). The higher order factor structure and gender invariance of the pathological narcissism inventory. Assessment, 17(4), 467–483. 10.1177/1073191110373227 20634422

[papt70056-bib-0054] Wright, A. G. C. , Stepp, S. D. , Scott, L. N. , Hallquist, M. N. , Beeney, J. E. , Lazarus, S. A. , & Pilkonis, P. A. (2017). The effect of pathological narcissism on interpersonal and affective processes in social interactions. Journal of Abnormal Psychology, 126(7), 898–910. 10.1037/abn0000286 29106275 PMC5679127

[papt70056-bib-0055] Zebhauser, A. , Hofmann‐Xu, L. , Baumert, J. , Häfner, S. , Lacruz, M. E. , Emeny, R. T. , Döring, A. , Grill, E. , Huber, D. , Peters, A. , & Ladwig, K. H. (2014). How much does it hurt to be lonely? Mental and physical differences between older men and women in the KORA‐age study. International Journal of Geriatric Psychiatry, 29(3), 245–252. 10.1002/gps.3998 23804458

